# Proteomic Stable Isotope Probing Reveals Taxonomically Distinct Patterns in Amino Acid Assimilation by Coastal Marine Bacterioplankton

**DOI:** 10.1128/mSystems.00027-15

**Published:** 2016-04-26

**Authors:** Samuel Bryson, Zhou Li, Jennifer Pett-Ridge, Robert L. Hettich, Xavier Mayali, Chongle Pan, Ryan S. Mueller

**Affiliations:** a Department of Microbiology, Oregon State University, Corvallis, Oregon, USA; b The University of Tennessee, Knoxville, Tennessee, USA; c Oak Ridge National Laboratory, Oak Ridge, Tennessee, USA; d Lawrence Livermore National Laboratory, Livermore, California, USA; University of Texas at El Paso

**Keywords:** environmental microbiology, marine microbiology, microbial communities, microbial ecology, proteomics

## Abstract

Heterotrophic marine bacterioplankton are a critical component of the carbon cycle, processing nearly a quarter of annual primary production, yet defining how substrate utilization preferences and resource partitioning structure microbial communities remains a challenge. In this study, proteomic stable isotope probing (proteomic SIP) was used to characterize population-specific assimilation of dissolved free amino acids (DFAAs), a major source of dissolved organic carbon for bacterial secondary production in aquatic environments. Microcosms of seawater collected from Newport, Oregon, and Monterey Bay, California, were incubated with 1 µM ^13^C-labeled amino acids for 15 and 32 h. The taxonomic compositions of microcosm metaproteomes were highly similar to those of the sampled natural communities, with *Rhodobacteriales*, SAR11, and *Flavobacteriales* representing the dominant taxa. Analysis of ^13^C incorporation into protein biomass allowed for quantification of the isotopic enrichment of identified proteins and subsequent determination of differential amino acid assimilation patterns between specific bacterioplankton populations. Proteins associated with *Rhodobacterales* tended to have a significantly high frequency of ^13^C-enriched peptides, opposite the trend for *Flavobacteriales* and SAR11 proteins. *Rhodobacterales* proteins associated with amino acid transport and metabolism had an increased frequency of ^13^C-enriched spectra at time point 2. *Alteromonadales* proteins also had a significantly high frequency of ^13^C-enriched peptides, particularly within ribosomal proteins, demonstrating their rapid growth during incubations. Overall, proteomic SIP facilitated quantitative comparisons of DFAA assimilation by specific taxa, both between sympatric populations and between protein functional groups within discrete populations, allowing an unprecedented examination of population level metabolic responses to resource acquisition in complex microbial communities.

**IMPORTANCE** An estimated 50 gigatons of carbon is annually fixed within marine systems, of which heterotrophic microbial populations process nearly half. These communities vary in composition and activity across spatial and temporal scales, so understanding how these changes affect global processes requires the delineation of functional roles for individual members. In a step toward ascertaining these roles, we applied proteomic stable isotope probing to quantify the assimilation of organic carbon from DFAAs into microbial protein biomass, since the turnover of DFAAs accounts for a substantial fraction of marine microbial carbon metabolism that is directed into biomass production. We conducted experiments at two coastal North Pacific locations and found taxonomically distinct responses. This approach allowed us to compare amino acid assimilation by specific bacterioplankton populations and characterize their allocation of this substrate among cellular functions.

## Abstract (precis)

An estimated 50 gigatons of carbon is annually fixed within marine systems, of which heterotrophic microbial populations process nearly half. These communities vary in composition and activity across spatial and temporal scales, so understanding how these changes affect global processes requires the delineation of functional roles for individual members. In a step toward ascertaining these roles, we applied proteomic stable isotope probing to quantify the assimilation of organic carbon from DFAAs into microbial protein biomass, since the turnover of DFAAs accounts for a substantial fraction of marine microbial carbon metabolism that is directed into biomass production. We conducted experiments at two coastal North Pacific locations and found taxonomically distinct responses. This approach allowed us to compare amino acid assimilation by specific bacterioplankton populations and characterize their allocation of this substrate among cellular functions.

## INTRODUCTION

Marine microbial communities are an essential component of global carbon cycles (1–4), but problems of scale and cultivability (5) have hindered progress in defining the functional roles of individual taxa within ecosystems. The development of culture-independent tools has revealed the phylogenetic and functional diversity of marine microbial communities and allowed for detailed observations of temporal and spatial population dynamics in natural environments (6–11). However, understanding how community diversity is maintained in a competitive environment, and relating the dynamics of specific populations to ecosystem processes remains a challenge. 

Our current understanding of the biogeochemical significance of marine bacterioplankton relies on decades of research on marine chemistry and measurements of bulk microbial community processes (12–17), with many studies focused on defining the role of dissolved free amino acids (DFAAs) as a substrate for heterotrophic bacteria (18–20). Turnover of bulk DFAAs can account for between 5 and 55% of total dissolved organic carbon (DOC) turnover in marine systems. Approximately 50% of the carbon from DFAAs utilized by microbes is assimilated (21, 22). While bulk measurements have illustrated the importance of heterotrophic bacterioplankton to the rapid utilization of this labile substrate, the specific contribution of individual populations toward DFAA turnover, as well as other DOC components, remains an area of active research. 

Experiments have examined the roles of individual bacterioplankton taxa in DOC turnover through indirect assessments of gene expression (11, 23–28) and direct observations of isotopically labeled substrate incorporation into cellular biomass. Several experimental techniques have been developed to determine taxonomically resolved carbon assimilation patterns for natural microbial populations. A combination of microautoradiography and fluorescence *in situ* hybridization (MAR-FISH) has been used to show amino acid and other types of DOC utilization by single marine bacterioplankton cells that are taxonomically identified with FISH probes (29–32). Another technique, DNA stable isotope probing (DNA-SIP), applies metagenomic approaches to whole-community DNA that is separated based on differences in density due to isotopic label incorporation into newly synthesized DNA (33–36). A third option, Chip-SIP, is a recently developed high-throughput method that quantifies isotopic label incorporation into RNA molecules that are hybridized to a microarray (37–39). 

A fourth technique measures stable isotope incorporation into protein biomass through the use of high-accuracy mass spectrometry (MS). Currently, there are two analytical approaches that differ in how isotopic labeled proteins are identified and how isotope incorporation is quantified. The protein-SIP approach produces detailed quantification of label incorporation into a defined set of identified proteins. First, unlabeled peptides are identified by tandem mass spectrometry (MS/MS), and then isotope incorporation into a predefined set of peptides is characterized through manual examination of mass spectra from coeluted isotopologues (40–44). The proteomic stable isotope probing (proteomic SIP) technique (45) used in this study expands on current metaproteomic approaches (46–48) by providing detailed information on stable isotope enrichment patterns for a wide diversity of peptides, in addition to identifying and quantifying thousands of unlabeled microbial proteins. Labeled peptide detection is made possible with the Sipros program (45, 49, 50), which finds peptide spectral matches (PSM) across a defined range and interval of percent ^13^C enrichments (e.g., 0 to 100% ^13^C at 1% increments in this study). Proteomic SIP has been applied with success to acid mine drainage biofilms to track activity related to ^15^N and ^2^H assimilation (45, 51). Here we report the application of proteomic SIP to quantify assimilation of ^13^C-labeled amino acids over two time points spanning 32 h by specific populations of bacterioplankton within samples of two coastal marine microbial communities.

The nature of the data generated from these experiments allowed us to establish two metrics for quantifying labeled substrate incorporation into detected proteins. The first metric, “label frequency,” is the relative frequency or proportion of PSM that are labeled (^13^C enrichment greater than naturally occurring levels) within the total set of PSM for an individual protein or for a taxonomic or functional group of proteins. Label frequency quantifies the breadth of *de novo* protein synthesis, but it does not discriminate between mechanisms of substrate incorporation, such as direct uptake and assimilation of a substrate, indirect incorporation from recycling of labeled biomass, or label incorporation from cross-feeding. The second metric, “average enrichment,” does provide a quantitative measure of labeled substrate incorporation into newly synthesized proteins. Average enrichment is the average percent ^13^C atom enrichment of the labeled PSM assigned to a given protein or group of proteins. Accordingly, high “average enrichment” denotes the direct assimilation of ^13^C-labeled substrate. Using these metrics, we quantified taxonomic differences in amino acid assimilation, showing general conservation among genera within the same taxonomic order. We also propose physiological mechanisms for these taxonomic differences based on differences in label frequency between groups of proteins associated with specific clusters of orthologous groups of proteins (COG) functional categories.

## RESULTS

### General metaproteomic results.

**FIG 1:**
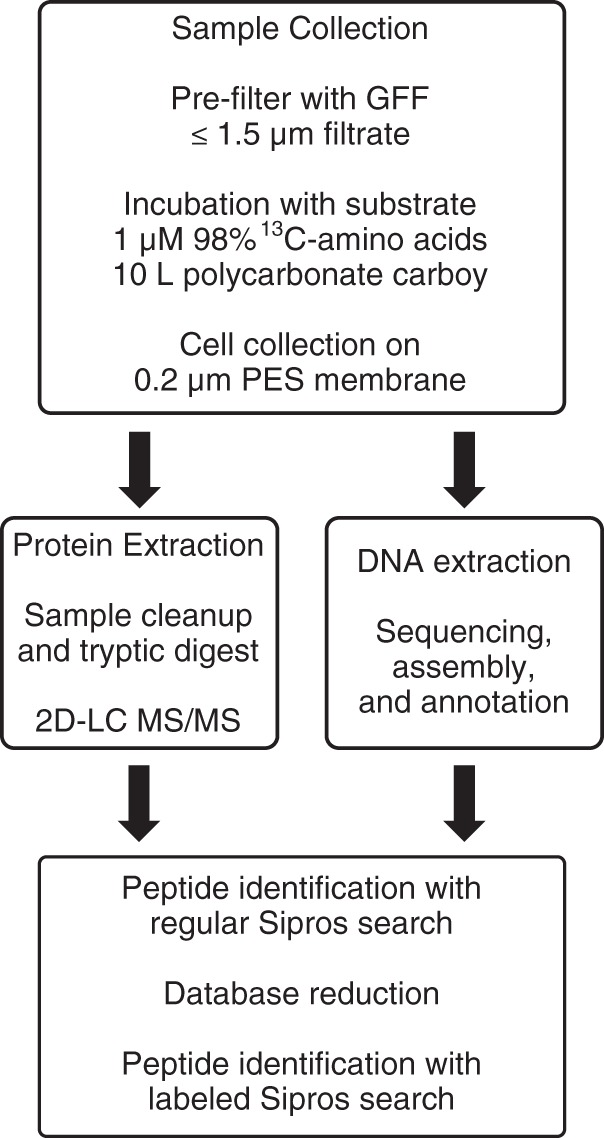
Overview of proteomic SIP experiment. GFF, glass fiber filters; PES, polyether sulfone.

An overview of our experimental approach is illustrated in Fig. 1 and briefly described here. Eight microcosm incubations were performed on samples from two locations. Two microcosms that contained surface water sampled from the Oregon coast, OR1 and OR2, were incubated with ^13^C-amino acids for 15 h and 32 h, respectively (time point 1 and time point 2, respectively). Six microcosms contained Monterey Bay (MB) surface water that was also incubated with the same substrate over the same two time points (MB1a, MB1b, and MB1c [each incubated for 15 h] and MB2a, MB2b, and MB2c [each incubated for 32 h]). At each time point, microcosm microbial communities were collected, and then proteins were extracted and analyzed using high-resolution tandem mass spectrometry. Sipros searches of mass spectra obtained for each microcosm were performed against a peptide matching database consisting of isolate marine microbial protein sequences and predicted coding sequences (CDS) from assembled metagenomes (52) from the Monterey Bay samples. The taxonomic affiliations of predicted CDS from the metagenome assembly and the peptide matching database are indicated in Fig. S1A in the supplemental material. Total PSM, peptide identifications, protein identifications, and statistics concerning ^13^C enrichment of PSM are detailed in Table 1.

10.1128/mSystems.00027-15.1 Figure S1  Taxonomic distribution of predicted open reading frames (ORFs), metagenome reads, and 16S rDNA amplicons. (A) Proportions of predicted ORFs for taxonomic groups in the assembled MB metagenomes and in the final database used for peptide spectral matching. (B) Distribution of mapped metagenomic reads among the assembled contigs for the initial MB0 sample and MB1. (C) Relative proportions of 16S rDNA amplicon taxonomic assignments for MB0, MB1, and MB2. Download Figure S1, EPS file, 0.3 MB. Copyright © 2016 Bryson et al. 2016 Bryson et al. This content is distributed under the terms of the Creative Commons Attribution 4.0 International license.

**TABLE 1:** Summary of metaproteomics results

Sample name	Sample location	Incubation time (h)	Peptide spectral matches	No. of peptides identified	No. of proteins identified	Label frequency (%)	Avg enrichment (%)
MB1a	Monterey Bay, CA	15	22,208	18,631	3,378	2.43	14.96
MB1b	Monterey Bay, CA	15	22,669	19,800	3,613	2.72	16.01
MB1c	Monterey Bay, CA	15	25,157	19,668	3,513	2.14	15.57

MB2a	Monterey Bay, CA	32	15,959	15,170	2,885	3.95	20.17
MB2b	Monterey Bay, CA	32	17,928	14,992	2,772	4.51	24.77
MB2c	Monterey Bay, CA	32	17,510	15,675	2,860	5.72	39.93

OR1	Newport, OR	15	6,554	6,441	1,190	9.22	33.04
OR2	Newport, OR	32	6,374	6,180	1,147	10.34	20.76

**FIG 2:**
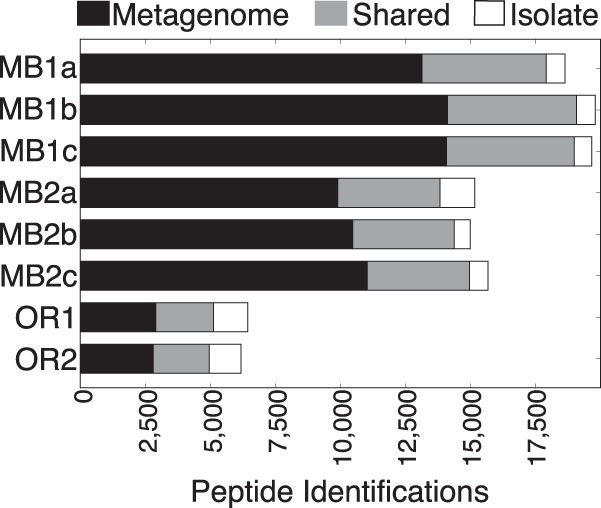
Peptide identifications by sequence source. “Metagenome” refers to peptides identified in predicted protein coding sequences (CDS) from an assembled metagenome from the Monterey Bay (MB) samples. “Isolate” refers to peptides identified in CDS from reference genomes. “Shared” refers to peptides found in CDS from both sources.

Substantially more peptide identifications came from metagenome-derived protein sequences than from isolate genome sequences (Fig. 2), even when the metagenome was not derived from the same sample as the metaproteome. Most of the peptides identified from isolate genome sequences were also identified in metagenomic protein sequences. The metagenome database performed better with the matched MB metaproteome samples than for the unmatched OR samples, for which no metagenome was available, yet nearly 60% of the proteins identified in the OR coast metaproteomes were from the MB metagenome sequences and not isolate genome sequences, many of which were collected from the Oregon coast (5). 

### Taxonomic and functional composition of metaproteomes and metagenomes.

The replicate proteomes from the Monterey Bay experiment were highly correlated at both time points, as evidenced by pairwise determination of the concordance correlation coefficient between the relative abundances of identified proteins defined by normalized balanced spectral counts (see Table S2 in the supplemental material). Within-replicate pairwise comparisons of MB1 and MB2 samples had average correlation scores of 0.82 and 0.71, respectively. The average correlation between all pairwise comparisons between time point 1 and time point 2 replicates was 0.66, indicating greater differences in measured protein abundances between time points than between replicates within a time point. The correlation between time points is comparable to the correlation coefficient for a comparison between OR1 and OR2 (ρ = 0.65). 

Shotgun metagenome sequencing (for time points 0 and 1, MB0 and MB1) and 16S rDNA sequencing (for MB0, MB1, and MB2) of the Monterey Bay sample indicated substantial population shifts that occurred between sample collection and subsequent time points. The relative abundances of taxonomic groups as determined by metagenomic read recruitment (see Fig. S1B in the supplemental material) and operational taxonomic unit (OTU) assignment of 16S rDNA amplicons (Fig. S1C) revealed a relative increase in *Alteromonadales* populations compared to a relative decline in the abundance of other initially low-abundance taxa during the first 15 h of incubation. Both sequencing data sets showed a relative increase in *Flavobacteriales* populations over the course of the incubations; however, this trend was not evident in the proteomic data where the relative abundance of mass spectra declined between MB1 and MB2 (Fig. S2). Overall, the taxonomic distribution of mass spectra in the metaproteomes resembled the composition of the sampled MB community as determined by DNA sequencing. 

10.1128/mSystems.00027-15.2 Figure S2  Comparison of metagenomes and metaproteomes. (A to C) Relative abundance of mapped reads from the MB0 and MB1 metagenomes (A), average relative spectral counts in MB1 and MB2 (B), and relative spectral counts in OR1 and OR2 (C). Download Figure S2, EPS file, 0.1 MB. Copyright © 2016 Bryson et al. 2016 Bryson et al. This content is distributed under the terms of the Creative Commons Attribution 4.0 International license.

**FIG 3:**
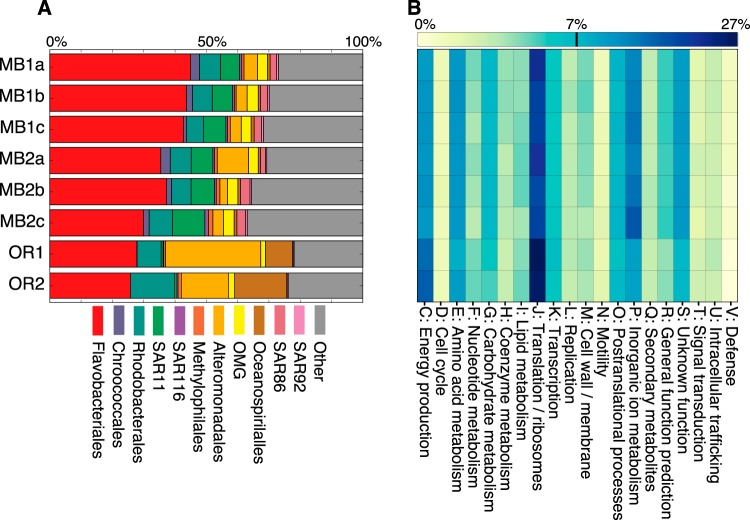
Relative abundance of protein identifications by taxonomy and COG functional category. (A) Stacked bar chart of proportion of total protein identifications in each sample for abundant orders of *Bacteria*. “Other” refers to low abundance and unclassified proteins, including identified eukaryote and *Archaea* proteins. (B) Heat map of proportion of protein identifications in each sample for general COG categories represented in the metaproteomes.

When proteins were assigned by taxonomy, observed differences in relative spectral counts (see Fig. S2 in the supplemental material) and total protein identifications (Fig. 3A) were greater between sampling locations than between time points. Proteins associated with the order *Flavobacteriales* represented a substantial proportion of total protein identifications in all samples, but relative counts declined from the first time point to the second time point in all of the samples. Total protein identifications for SAR11 were substantially lower in the OR samples (8 and 9 in OR1 and OR2) than in the MB samples (average numbers of 231 and 234 in MB1 and MB2); in both locations, most of these proteins were assigned to the coastal Oregon isolate HTCC1062 (53). The relative contribution of *Rhodobacterales* protein identifications was similar between the two locations, but absolute counts were lower in the OR samples (91 and 161 in OR1 and OR2 and average numbers of 231 and 234 in MB1 and MB2). *Alteromonadales* proteins contributed a substantially greater fraction of the community proteome in the two OR samples, but absolute numbers of these protein identifications were similar between the locations. Other *Gammaproteobacteria* clades, such as members of the OMG, SAR86, and SAR92 clades, had relatively higher numbers of protein identifications in the MB proteomes. Conversely, *Oceanospirillales* protein assignments accounted for less than 1% of protein identifications in the six MB samples versus 8.7% and 16.7% of identified proteins in OR1 and OR2. 

We determined the proportion of identified proteins assigned to COG categories (Fig. 3B) and found negligible differences between the functional profiles of the bacterioplankton communities at both locations. The most frequently detected proteins were assigned to COG category J (translation and ribosomal structure and biogenesis), followed by proteins belonging to categories C (energy production and conversion), P (inorganic ion transport and metabolism), E (amino acid transport and metabolism), G (carbohydrate transport and metabolism), and S (unknown function). Compared to the six MB proteomes, a higher proportion of protein identifications in the two OR proteomes were assigned to COG categories J and C, while a lower portion of proteins were assigned to categories P and S. 

### Community level assimilation of amino acids determined by ^13^C labeling of proteomes.

**FIG 4:**
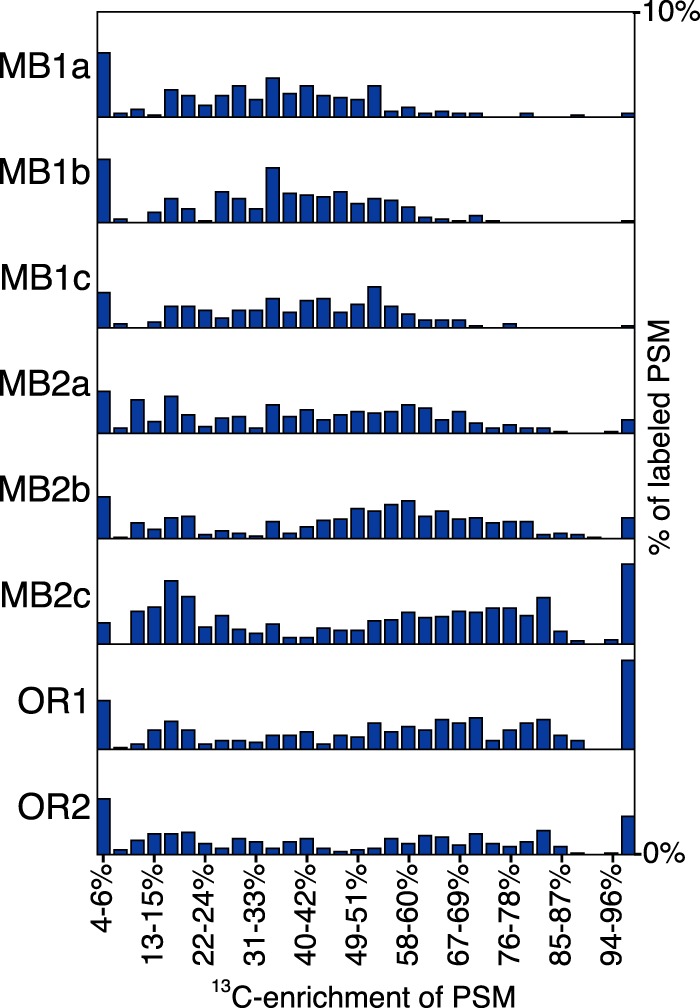
Histogram of highly labeled peptide spectral matches (PSM). The histogram depicts the proportion of labeled spectra (^13^C enrichment of ≥2%) from 4% to 99% ^13^C enrichment within 3% enrichment bins. Each distribution depicts bars on a relative scale of 0 to 10% of total labeled spectra. Label frequency and average enrichment values for the samples are presented in Table 1.

We analyzed the ^13^C enrichment of all samples in order to assess community level amino acid assimilation with respect to both location and incubation time. We observed significant changes in both the label frequency (*P* = 0.01 by *t* test) and average enrichment (*P* = 0.05 by *t* test) in the MB samples between time points 1 and 2, while there was little change between OR1 and OR2 (Fig. 4 and Table 1). At the first time point, the three MB1 samples exhibited similar label frequency (average frequency of 2.4% ± 0.29% standard deviation [SD]) and average enrichment (average frequency of 15.5% ± 0.53% SD), but the three MB2 proteomes exhibited greater variability in label frequency (average frequency of 4.7% ± 0.90% SD) and average enrichment (average frequency of 28.3% ± 10% SD). The patterns of ^13^C enrichment in the MB2 proteomes were more similar to the OR1 and OR2 proteomes than the MB1 proteomes, as reflected by the histogram of labeled spectra (Fig. 4). The three MB1 samples had unimodal distributions centered around 35% ^13^C enrichment, whereas the distributions of labeled spectra from the three MB2 samples and the two OR samples were more representative of a bimodal distribution, with a lower peak at 16 to 18% and the second peak above 50% ^13^C enrichment (Fig. 4), suggesting that incubation time increased the heterogeneity in label incorporation across populations.

The change in overall label frequency at both locations was positively and significantly correlated to the change in overall cell counts (Pearson’s *r* = 0.89). Average cell counts from the OR samples indicated that the microbial community increased nearly 3-fold during the first 15 h but increased by only 32% in the subsequent 17 h (counts of 0.496 × 10^6^, 1.48 × 10^6^, and 1.96 × 10^6^ cells ml^−1^, respectively). Average cell counts from the samples from Monterey Bay, California, suggest a higher initial population size (2.16 × 10^6^ cells ml^−1^), which increased by 8% in the first 15 h and again by 26% in the following 17 h (2.33 × 10^6^  and 2.94 × 10^6^ cells ml^−1^, respectively). Unlike label frequency, average enrichment was not significantly correlated with change in cell counts (Pearson’s *r* = 0.10); in the OR samples, average enrichment actually decreased between time points. 

### Taxonomic differences in amino acid assimilation.

**FIG 5:**
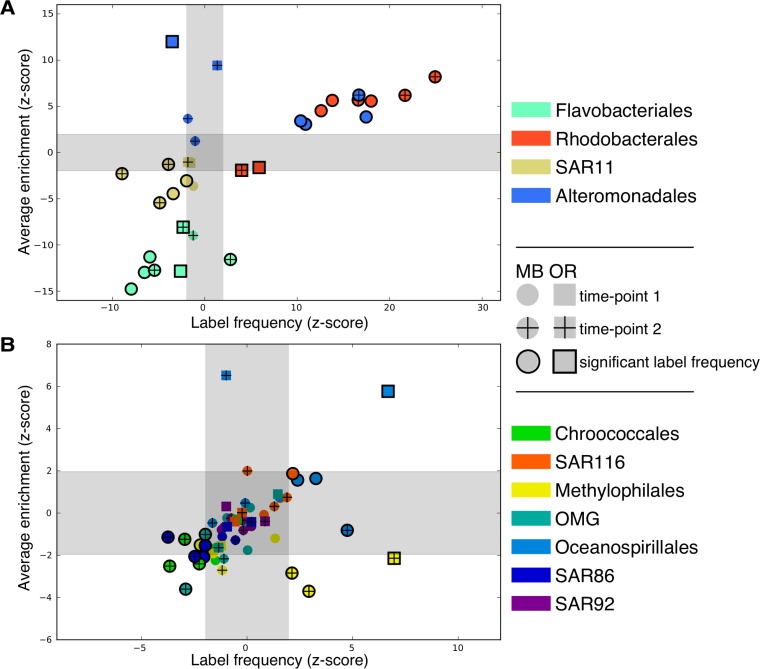
Label frequency and average enrichment of taxa. (A and B) more-represented (A) and less-represented (B) orders in the metaproteomes. The z-scores of label frequency (*x* axis) and average enrichment (*y* axis) based on comparisons of observed values with distributions under the null model are shown. The shaded area represents the inner 95% of values under a standard normal distribution. Significance testing is described in Materials and Methods.

Incorporation of amino acids into proteins was not limited to specific taxa; labeled PSM were detected for all taxonomic groups with greater than 12 protein identifications in any one metaproteome. To test the null hypothesis that all taxa could equally assimilate ^13^C-amino acids, we compared each taxon’s observed label frequency and average enrichment values calculated from each taxon’s proteome against a null distribution consisting of label frequency and average enrichment values for 1,000 randomly selected subsets with equivalent numbers of balanced spectral counts (Fig. 5). This null distribution, created independently for each test, represented the expected distribution of label frequency and average enrichment values if taxonomic labels were randomly assigned to PSM. Significant values were defined as the values more extreme than the inner 95% of expected values. Z-scores for each value presented in Fig. 5 are derived from each null distribution. For brevity, we focus on the most abundant taxa, namely, those within the *Proteobacteria* and *Bacteroidetes* phyla.

*Flavobacteriales* and SAR11 showed label frequency and average enrichment values that were significantly low compared to the null distribution in nearly all of the samples from both locations (Fig. 5A). Genus level assignment of proteins revealed a consistent trend in labeling for *Flavobacteriales*, with the notable exception of the genus *Flavobacterium* which had high label frequency in the OR and MB2 samples (see Fig. S3C in the supplemental material), while also supplanting *Polaribacter* as the *Flavobacteriales* genus with the most protein identifications in the MB2 samples. Average enrichment of *Alteromonadales* proteomes were significantly high in all samples except MB2b, and label frequency was significantly high in the three MB1 samples and in MB2c, but not in the OR samples (Fig. 5A). This trend was consistent at the genus level where label frequency was generally higher at time point 1 and average enrichment was generally high at both time points (Fig. S3B). In all eight metaproteomes, *Rhodobacterales* proteins had significantly high label frequency that increased between time points in the MB samples, with average enrichment significantly high in the MB samples, but not in the two OR samples (Fig. 5A). The labeling of genera within the *Rhodobacterales* was generally consistent, but with a wide range of label frequency values from insignificant to significantly high (Fig. S3A).

10.1128/mSystems.00027-15.3 Figure S3  Label frequency and average enrichment for genera of three abundant orders, *Rhodobacterales* (A), *Alteromonadales* (B), and *Flavobacteriales* (C). The figure depicts Z-scores of label frequency (*x* axis) and average enrichment (*y* axis) based on comparisons of observed values with distributions under the null model. The shaded area represents the inner 95% of values under a standard normal distribution. Significance testing is described in Materials and Methods. Download Figure S3, EPS file, 0.7 MB. Copyright © 2016 Bryson et al. 2016 Bryson et al. This content is distributed under the terms of the Creative Commons Attribution 4.0 International license.

*Oceanospirillales* proteins had high average enrichment at both OR time points, whereas label frequency varied between samples (Fig. 5B). Proteins from SAR92 bacteria were insignificantly labeled in all but one sample, MB1b, where both label frequency and average enrichment values were significantly low (Fig. 5B). *Gammaproteobacteria* proteins from the SAR86 and OMG clades had labeling that was generally insignificant or low across all samples (Fig. 5B). The SAR116 proteome was significantly high in label frequency in MB1b, MB1c, and MB2b, but its average enrichment was significantly high only in MB2c (Fig. 5B). Average enrichment values for the *Methylophilales* proteome were significantly low in five of the samples, but label frequency was significantly high in two MB2 samples and in OR2 (Fig. 5B). *Chroococcales* proteins were significantly low in labeling in most samples (Fig. 5B).

In addition to assessing differential substrate assimilation between taxa, our experiment permitted the assessment of label frequency for proteins assigned to different COG functional categories within specific taxonomic groups. Significance testing was conducted in a similar manner as the previously described tests for differential ^13^C enrichment of taxonomic groups. Here the null distribution assumes that ^13^C enrichment of proteins within a taxonomic group is independent of COG category label, i.e., all types of proteins should have equal label frequency. By comparing the observed label frequency for each COG category to the values expected under the null distribution, we can assess the physiological basis for observed taxonomic differences in ^13^C enrichment. The results of this analysis show that the *Alteromonadales* proteomes had significantly high label frequency in COG category J (translation) in all six MB samples (see Fig. S4B in the supplemental material). This is in contrast to the *Flavobacteriales* and *Rhodobacterales* proteomes in which label frequencies were significantly high for COG category J at MB1, but significantly lower than expected at MB2 (Fig. S4A and S4C). *Flavobacteriales* proteins in COG category P (inorganic ion transport and metabolism) had significantly high labeling in all time point 2 proteomes. The *Rhodobacterales* proteome had increased labeling in COG category E (amino acid transport and metabolism) in the MB2 samples and both OR samples. Additionally, this taxonomic order had significantly low labeling of carbohydrate metabolism proteins (COG category G) in five of the six MB proteomes. Both SAR11 and *Rhodobacterales* had consistently high labeling of proteins associated with protein modification, turnover, and chaperones (COG category O).

10.1128/mSystems.00027-15.4 Figure S4  Relative labeling of COG categories. The panels depict biclustered heat maps of label frequency (Z-scores) of COG functional categories for each sample within three dominant orders: *Rhodobacterales* (A), *Alteromonadales* (B), and *Flavobacteriales* (C). Download Figure S4, EPS file, 0.3 MB. Copyright © 2016 Bryson et al. 2016 Bryson et al. This content is distributed under the terms of the Creative Commons Attribution 4.0 International license.

## DISCUSSION

### Microbial community activity varies between time and location.

Variability among replicates and geographic location allows us to speculate on the physiological responses of different populations that could be driving the observed patterns of ^13^C enrichment. In this study, we found that the MB2 samples and the OR1 sample had similar label frequency and average enrichment values, suggesting more rapid DFAA assimilation in the OR samples than in the MB samples. This observation could result from a substantially greater fraction of cells being in an active state and ready to assimilate the amended amino acids in the OR samples; total cell numbers increased relatively more in the OR samples than the MB samples within the time frame of the experiment. Alternatively, the OR samples could have included greater initial populations of taxa with the capacity for rapid amino acid assimilation, as evidenced by the greater relative numbers of protein identifications and mass spectra associated with two highly labeled *Gammaproteobacteria* populations, *Alteromonadales* and *Oceanospirillales*. The OR2 samples had increased label frequency and decreased average enrichment relative to the OR1 samples, suggesting that the pool of labeled proteins had undergone greater turnover and the signal had been diluted among greater numbers of proteins. The apparent bimodal distribution of labeled spectra in MB2 and OR1 samples can indicate heterogeneity among community members with respect to amino acid assimilation representing both moderately and highly ^13^C-enriched populations. By testing and defining assimilation patterns for distinct taxonomic groups, amino acid utilization preferences were determined for the abundant microbial community members. Assessment of substrate assimilation for less abundant populations was also possible, including the findings of low DFAA assimilation by SAR86, which was also found in a previous MAR-FISH study (32). 

### Distinct populations differ in amino acid assimilation.

The relative dominance of *Flavobacteriales* proteins in the coastal samples is consistent with the results of other studies (29, 54–57). The two OR samples in this study exhibited higher relative abundances of *Flavobacteriales* mass spectra compared to a previous OR coast metaproteome (27); this could be due in part to better representation of proteins owing to inclusion of metagenomic data in the database. The significantly low relative assimilation of DFAAs by *Flavobacteriales* populations is similar to findings in a previous MAR-FISH study (29). Substrate concentration could have affected the overall activity of this population with regard to the low label frequency observed compared to other taxa. A Chip-SIP study that tracked ^15^N incorporation of DFAAs in San Francisco Bay found that members of the *Bacteroidetes* incorporated relatively more substrate with increasing substrate concentration (39), suggesting that this population might have been more highly labeled in our experiments if the amended substrate concentration had been greater. Experimental conditions removed particulate matter of ≥1.5 µm in size, potentially removing an important resource for *Flavobacteriales* populations, which are often identified as particulate colonizers and degraders responding initially to the presence of increased particulate matter occurring during phytoplankton blooms (11, 58). The observed relative decrease in protein identifications over the course of the experiment would seem to confirm this hypothesis; however, metagenomic sequence libraries (see Fig. S1B in the supplemental material) and 16S sequence libraries (Fig. S1C) provide evidence for an actively growing population, suggesting that alternative unlabeled substrates were utilized. The observed high label frequency but low average enrichment for proteins assigned to the genus *Flavobacterium* in the MB2 replicates (Fig. S3C) could be interpreted as a cross-feeding event in which this population utilized labeled by-products generated from DFAA metabolism of other community members.

In both the OR and MB proteomes, SAR11 proteins had significantly low label frequency that was even lower relative to expected values at time point 2, indicating relatively lower DFAA assimilation compared to the general community. This relative decline was also observed in the metagenome sequence libraries for the first 15 h of incubation but was not observed in the 16S amplicon data, as the primers used here have been reported to miss this clade (59). The observed low label frequency in this experiment differs from a prior MAR-FISH study that found SAR11 cells were highly represented in the fraction of cells actively assimilating DFAAs (31). However, substrate concentrations were much lower in that study (0.5 nM), suggesting that differences in observations may be based on an advantage of SAR11 cells in competing for substrates at low concentrations, and potentially being inhibited at high concentrations. The observed high label frequency in SAR11 proteins assigned to COG category O is congruent with previous findings of highly expressed protein maintenance and recycling genes in Sargasso Sea samples (28). The significant investment of resources into proteome maintenance provides insight into the observed low growth rate of SAR11 (53) which would accompany an overall low relative label frequency. Alternatively, increased expression of chaperones and heat shock proteins could represent a general stress response. In a previous study of transcriptional responses to bottle incubations, SAR11 was found to have increased expression of stress response genes and decreased expression of transporters (60). Although a previous metaproteome study of coastal Oregon (27) identified substantially more SAR11 proteins than these two OR proteomes, samples in this study were taken from waters adjoining an estuary. The relative abundance of SAR11 has been observed to increase with distance from near-shore bloom conditions, opposite the trend observed for populations of *Flavobacteriales* and *Rhodobacterales* (61). 

Populations of the metabolically versatile *Rhodobacterales* order have been shown to play important roles in the processing of carbon in coastal environments (26, 57, 62–65). This lineage has also been associated with the utilization of low-molecular-weight DOC, specifically DFAAs (30, 39). Our assimilation data support these hypotheses, as *Rhodobacterales* proteins were highly labeled in samples from both coastal locations and generally increased in both labeling metrics between time points. Increased relative abundance in both the metagenomes (see Fig. S1B in the supplemental material) and 16S rDNA amplicon libraries during the first 15 h of incubation also indicates an active population. The proteomic SIP results are similar to findings that ^15^N incorporation from DFAAs by this taxon was similar at both high (5 µM) and medium (500 nM) concentrations (39), as depletion of the labeled substrate would be expected over the course of the incubation. Indeed, the observed high label frequency for *Rhodobacterales* amino acid metabolism proteins in the time point 2 samples suggests that this population responded to depleted DFAAs by building more machinery to acquire this substrate. The moderate percent ^13^C enrichment values observed also fit with previous Chip-SIP observations of 25% higher N-use efficiency than C from DFAAs (38), suggesting the role of DFAAs as a significant source of N. Additionally, *Rhodobacterales* represented a substantial fraction of the initial sampled MB community (Fig. S1B and S1C), which if active would assimilate labeled substrate across a greater number of cells, resulting in low to moderate average enrichment values compared to a population of new cells with newly synthesized proteins composed of a greater proportion of newly acquired labeled substrate.

Previous work examining marine community responses to dissolved organic matter and deep seawater amendment have characterized members of the *Alteromonadales* order as fast-growing copiotrophic organisms (66, 67). In this study, label frequency and average enrichment values indicated that this population assimilated a higher percentage of ^13^C from labeled DFAAs into protein biomass than other taxa, suggesting rapid initial growth in response to the incubation conditions that was confirmed by sequencing data (see Fig. S1B and S1C in the supplemental material). At time point 2, there was greater variability in the labeling of this taxon and in the relative abundance of 16S OTUs assigned to this taxonomic order. In most samples, their label frequency declined while average enrichment remained high, concurrent with the decline in relative abundance in 16S libraries. This could be due to continued, less rapid growth on unlabeled substrates with limited turnover of early synthesized proteins, specifically ribosomes and translational machinery, where these bacteria are possibly less competitive for amino acid uptake at lower concentrations. Consistent with this hypothesis, Chip-SIP experiments found that *Alteromonadales* increased labeling with increased substrate concentration (39). Unlike the *Flavobacteriales* and *Rhodobacterales* proteomes, there was not a distinction between time points for labeling of COG categories. Instead, this population consistently devoted labeled substrate to translational machinery and energy production proteins, with little apparent turnover or dilution of the label signal. 

## MATERIALS AND METHODS

### Sample collection, processing, and substrate incubation experiments.

Oregon coast samples (OR) were collected in Newport, OR (44°37.069′N, 124°3.455′W, ~0 km from shore), on 16 July 2013 at 5:00 p.m. during incoming high tide. Monterey Bay (MB) samples were collected from surface waters (36°53.387′N, 121°57.257′W, ~10 km from shore) while on board the RV *Rachel Carson* on 14 October 2013. All samples were prefiltered using a Geotech polycarbonate filter holder through 142-mm Whatman 934-AH glass fiber filters (GFF) (autoclaved and rinsed with distilled water) with a nominal retention size of 1.5 µm. Microcosm incubations of filtrate were carried out in acid-washed and rinsed 10-liter polycarbonate carboys, amended with a final concentration of ~1 µM 98 atom% ^13^C-labeled algal amino acids (catalog no. 426199-1G; Sigma-Aldrich). Incubations were performed at constant temperatures close to the temperature of the ambient water column (16°C for OR samples and 19°C for MB samples) for 15 and 32 h. Upon harvest, cells from replicate microcosms were concentrated in parallel on 0.2-µm polyethersulfone (PES) membrane filters (42 mm; Pall) using a peristaltic pump, Pall 42-mm polycarbonate in-line filter holders, and 42-mm GFF support filters. Filters were changed as needed for maintaining a consistent flow of ~100 ml min^−1^. Upon completion, membrane filters were transferred to sterile 15-ml tubes, immediately frozen on dry ice, and subsequently transferred to a −80°C freezer for long-term storage. For all MB samples, 1 liter of retentate was reserved for DNA extraction and subsequent metagenome sequencing as described by Mueller et al. (52). Formaldehyde-fixed and SYBR green-stained cells were counted with a Guava Technologies flow cytometer as previously described (68, 69).

### Protein extraction, purification, digestion, and mass spectrometry.

Protein was extracted from cells concentrated on membrane filters as follows. First, membrane filters were cut into thin strips and placed in sterile 15-ml tubes. Next, 3 ml of MoBio solution ST1B was added, and tubes were vortexed. Three milliliters of sodium dodecyl sulfate (SDS) lysis buffer plus dithiothreitol (DTT) was then added to each sample, and the tubes were vortexed and subsequently incubated for 15 min at 90°C. Cell lysate was transferred to new 2-ml Eppendorf Protein LoBind tubes, and cellular debris was removed by centrifugation. Supernatant from the tubes was transferred to new 2-ml Protein Lo Bind tubes, and protein was precipitated overnight with trichloroacetic acid (TCA). The concentration of protein in extracts was quantified using the Qubit protein assay kit (Invitrogen). Extractions from microbial cells collected from 9 liters of seawater typically yielded ~200 µg of purified protein, which was sufficient for full mass spectrometry runs requiring 50 to 100-µg protein digest.

Protein pellets were resolubilized in 6 M guanidine and 10 mM DTT. Then, 50 µg of protein was further cleaned up and digested by modified trypsin on centrifugal filters with a 30,000-molecular-weight cutoff (70, 71). Each sample was first digested overnight at an enzyme/substrate ratio of 1:100 (weight/weight) at room temperature with gentle shaking, followed by a secondary digestion for 4 h. For each sample, 25 µg of peptides was loaded offline into a 150-µm-inner-diameter (ID) two-dimensional (2D) back column (Polymicro Technologies) packed with 3 cm of C_18_ reverse-phase (RP) resin (Luna; Phenomenex) and 3 cm of strong-cation-exchange (SCX) resin (Luna; Phenomenex). The back column loaded with peptides was desalted offline with 100% solvent A (95% H_2_O, 5% acetonitrile, and 0.1% formic acid) and washed with a 1-h gradient from 100% solvent A to 100% solvent B (30% H_2_O, 70% acetonitrile, and 0.1% formic acid) to move peptides from RP resin to SCX resin. The back column was then connected to a 100-µm-ID front column (New Objective) packed in-house with 15 cm of C_18_ RP resin and placed in-line with a U3000 quaternary high-performance liquid chromatography (HPLC) pump (Dionex). Multidimensional protein identification technology (MudPIT) was used for the liquid chromatography coupled to tandem mass spectrometry (LC-MS/MS) measurements (72). Each mass spectrometric run was configured with 11 SCX fractionations using 5%, 7%, 10%, 12%, 15%, 17%, 20%, 25%, 35%, 50%, and 100% of solvent D (500 mM ammonium acetate dissolved in solvent A). Each SCX fraction was separated by a 110-min RP gradient from 100% solvent A to 50% solvent B. The LC eluent was directly nanosprayed (Proxeon) into an LTQ Orbitrap Elite mass spectrometer (Thermo Scientific). Both MS scans and collision-induced dissociation (CID)MS/MS scans were acquired in Orbitrap with a resolution of 30,000 and 15,000, respectively. After each MS scan, the eight most abundant precursor ions were selected under automated data-dependent acquisition for MS/MS analysis by CID. 

### Metaproteome database construction, spectra searches, and label quantification.

Given that sample-specific metagenomes were not available for OR samples, multiple databases comprised of protein sequences from isolate marine microbes and metagenomes generated for the MB samples were constructed for confident identification of peptide spectral matches (PSM) with the Sipros program (45, 49, 50). All searches considered tryptic peptides in the range of 6 to 60 amino acids with a maximum of two missed cuts. Parent ion and fragment ion mass tolerances were 0.05 Da and 0.02 Da, respectively. Regular searches considered only natural ^13^C abundance (1.109%). SIP searches considered ^13^C enrichments of 0% to 100% in 1% increments. A peptide level 1% false-discovery rate (FDR) using decoy sequences was applied in both regular and SIP searches. The two-peptide rule, one unique plus one shared peptide identification, was required for protein identifications. All Sipros searches were performed on the Titan supercomputer.

In order to determine whether peptide mass spectra from a known unlabeled proteome were falsely identified as being enriched with ^13^C label, the spectra from two unlabeled coastal marine samples were searched with Sipros in SIP enrichment-searching mode. The FDR for spectra that were incorrectly assigned enrichment values above the natural abundance of ^13^C (≥2%) averaged 2.1%. No spectra were falsely assigned ^13^C enrichment greater than 7% (see Table S1 in the supplemental material). 

10.1128/mSystems.00027-15.5 Table S1  False detection rate of labeled spectra. The table lists numbers of and proportion of total PSM from two unlabeled metaproteomes that were identified at the corresponding level of percent ^13^C enrichment. Searches were conducted against the same sequence database used for SIP searches in this study. Download Table S1, PDF file, 0.04 MB. Copyright © 2016 Bryson et al. 2016 Bryson et al. This content is distributed under the terms of the Creative Commons Attribution 4.0 International license.

10.1128/mSystems.00027-15.6 Table S2  Pairwise concordance correlation coefficients. Results are from pairwise tests between all metaproteomes using relative balanced spectral counts for each identified protein. Download Table S2, PDF file, 0.03 MB. Copyright © 2016 Bryson et al. 2016 Bryson et al. This content is distributed under the terms of the Creative Commons Attribution 4.0 International license.

Comparisons between individual search outputs of multiple database configurations were made to evaluate the influence of database design on protein identification results. Regular searches of the OR samples were performed against an initial database of protein coding sequences (CDS) from 133 available marine bacteria and archaea genomes. Subsequent searches queried a smaller database of CDS from 36 of these genomes with the most protein identifications. A separate database of CDS was also created from an assembly of metagenomes from MB samples (52), which included sequences from pooled DNA extracted from the three MB1 replicates in this study. Reads from MB metagenomes were assembled using MetaRay, version 2.3.1 (73). The resulting contigs were clustered at 99% sequence identity with cd-hit-est (74) and merged with Minimus2 (75). Then, CDS were predicted using Prodigal (76). The taxon of each CDS was assigned based on the respective best hit against GenBank RefSeq (downloaded 14 June 2014) protein sequences using the Diamond search algorithm in “blastp” mode with default settings (77). Functional assignments of individual coding sequences were made against the eggNOG database, v.4.0 (78), based on respective best scoring matches against hidden Markov model (hmm) profiles of bacterial cluster alignment (bactNOG) protein families using HMMSCAN (79). Metagenome abundance abundances (see Fig. S1B in the supplemental material) were determined by mapping sequencing reads back to the assembled contigs using Bowtie 2 (80).

Each database, isolate genome CDS or assembled metagenome CDS database, was used for regular PSM searches of all eight samples considered in this study. In preliminary database search tests, no SIP searches identified labeled peptides (^13^C enrichment of ≥2%) that were not also identified in regular searches (data not shown). This allowed us to reduce the protein database for SIP searches to include only proteins with peptide identifications in any of the eight regular searches (search results in Table 1; taxonomic distribution of CDS in the metagenome and the final database used for searches in Fig. S1A in the supplemental material). 

### 16S rDNA amplicon library preparation and analysis.

For all MB1 and MB2 replicates plus three time point 0 (MB0) replicates, the extracted whole-community DNA (52) was subjected to PCR amplification of the v4 rRNA gene region using previously described dual indexed primers (81). Two sets of PCRs (technical replicates using two sets of barcodes) were carried out in triplicate using 25-µl volume, ~1-ng template, 20 cycles, and the Thermo Phusion high-fidelity PCR kit. Triplicate reaction mixtures were pooled, size selected, excised from 1% agarose gel, purified with the PureLink Quick gel extraction kit (Invitrogen), and then quantified with Qubit dsDNA (double-stranded DNA) BR assay kit (Thermo Fisher). Libraries were sequenced on the Illumina MiSeq platform at the Oregon State University Center for Genome Research and Biocomputing using the MiSeq Reagent kit v2 generating two 250-bp reads. Reads were quality filtered using Sickle (version 1.33; N. A. Joshi and J. N. Fass, 2011; available at https://github.com/najoshi/sickle), a minimum quality score of 25, and a minimum length of 150. Subsequent assembly, quality filtering, unsupervised OTU picking at 97% identity, taxonomic assignment with alignment to the Silva database (82), and relative abundance profiles were conducted in the mothur software package (83).

### Metaproteome data processing and statistical analyses.

Label incorporation into the peptides and proteomes of distinct taxonomic groups from each community were defined using two separate metrics: “label frequency” (the proportion of PSM with ^13^C content of ≥2%) and “average enrichment” (the average percentage of ^13^C in labeled PSM). Both metrics were applied to define labeling for the PSM associated with a set of proteins from a specific sample, from a specific taxon within a sample, or from a COG functional category within a specific taxonomic grouping. Significance testing for label frequency and average enrichment was conducted using a nonparametric approach in order to overcome differences in total detected label between samples and total PSM for proteins associated with taxonomic groups within a sample or COG functional categories within a taxon. For each subset of proteins that was tested, 1,000 permutations of equivalent numbers of spectra were randomly selected in order to define the distribution of expected values for label frequency and average enrichment. The null distribution for tests comparing taxonomic groups within a sample and tests comparing COG functional categories within a taxonomic group were drawn from each sample’s total spectra and from each taxon’s associated spectra within a sample, respectively. We defined significant observations as those that were as extreme or more extreme than the critical values delineating the inner 95% of values obtained by random permutations. The mean and standard deviation of each random distribution were used to define Z-scores for illustration purposes.

### Conclusions.

In this study of two North Pacific coastal microbial communities, we used proteomic SIP to identify thousands of proteins within each sample, providing access to the expressed genome of many of the diverse populations of planktonic cells (≤1.5 µm) within these communities. While we identified more than 1,000 proteins for each of the OR samples without the use of a matched metagenome, comparable to numbers found in previous marine metaproteomic experiments (47), the use of a matched metagenome database with the MB samples resulted in an approximately 2- to 3-fold increase in protein identifications. By extending metaproteomics with the application of ^13^C-based proteomic SIP, we quantified DFAA assimilation patterns among discrete populations and compared *de novo* protein synthesis between protein functional groups. 

Using proteomic SIP data along with comparisons of shifts in relative abundance from 16S rDNA amplicon sequencing and metagenomics provided additional insight into community dynamics that would remain obscured if only one technique was utilized. Proteomic SIP data indicated that *Flavobacteriales* populations had low average enrichment over the course of the incubations but did exhibit increases in both label frequency and relative abundance in sequence libraries; taken together, these provide compelling evidence for an active community potentially obtaining label through cross-feeding. Sequencing data also helped differentiate two populations with high label frequency. *Rhodobacterales* were an abundant component of the initial sampled community; while their proteomes consistently demonstrated high label frequency across samples and time points, their relatively moderate average enrichment suggested ^13^C-amino acid assimilation across an extant active population. Conversely, the high average enrichment of *Alteromonadales* populations indicated rapid ^13^C-amino acid conversion into newly synthesized cells, which was corroborated by increased relative abundance in the sequencing data. 

Analysis of DFAA assimilation across protein functional categories by *Rhodobacterales* and *Alteromonadales* during the second half of the incubation provided insight into these observed differences in population level responses. Natural communities experience substrate availability that is affected by rates of production and uptake, interpopulation competition, and substrate lability, all of which interact to shape the community. Observed increases in label frequency and relative abundance for *Rhodobacterales* were accompanied by significant synthesis of amino acid uptake and metabolism proteins, potentially conferring a competitive advantage for continued uptake of this substrate. Conversely, the initial rapid increase in the relative abundance of *Alteromonadales*, concurrent with ribosomal protein biosynthesis, did not continue during the second time period during which time available DFAAs were likely depleted.
