# Supplementary material for: Proteomic Stable Isotope Probing Reveals Taxonomically Distinct Patterns in Amino Acid Assimilation by Coastal Marine Bacterioplankton
Source: mSystems. 2016 Apr 26;1(2):e00027-15. doi: 10.1128/mSystems.00027-15 (PMC5069745; doi:10.1128/mSystems.00027-15)
Supplement: Table S1 [file sys002162015st5.pdf]

Table S1: False Detection Rate of Labeled Spectra

Unlabeled Sample: Newport, OR		
% ^{13}C-enrichment	PSM count	% total
0	179	0.431%
1	40,149	96.589%
2	909	2.187%
3	244	0.587%
4	70	0.168%
5	15	0.036%
6	0	0.000%
7	1	0.002%
8-100	0	0.000%
**Unlabeled Sample: Monterey Bay, CA**		
% ^{13}C-enrichment	PSM count	% total
0	67	0.244%
1	27,038	98.585%
2	285	1.039%
3	14	0.051%
4	19	0.069%
5	3	0.011%
6-100	0	0.000%
