# Supplementary material for: Proteomic Stable Isotope Probing Reveals Taxonomically Distinct Patterns in Amino Acid Assimilation by Coastal Marine Bacterioplankton
Source: mSystems. 2016 Apr 26;1(2):e00027-15. doi: 10.1128/mSystems.00027-15 (PMC5069745; doi:10.1128/mSystems.00027-15)
Supplement: Table S2 [file sys002162015st6.pdf]

Table S2: Pairwise Concordance Correlation Coefficients

sample	OR1	OR2	MB1a	MB1b	MB1c	MB2a	MB2b	MB2c
OR1		0.653	0.088	0.083	0.097	0.136	0.185	0.111
OR2			0.123	0.117	0.136	0.181	0.252	0.162
MB1a				0.831	0.819	0.623	0.702	0.618
MB1b					0.807	0.582	0.697	0.631
MB1c						0.624	0.746	0.722
MB2a							0.739	0.631
MB2b								0.755
MB2c								
